# Nutritional Knowledge, Dietary Behaviours and Supplementation Practices Among Pregnant Women in Poland: A Repeated Cross-Sectional Study Across a Decade

**DOI:** 10.3390/nu18142260

**Published:** 2026-07-10

**Authors:** Marzena Strahl, Eliza Wasilewska, Ewelina Chawłowska, Sylwia Małgorzewicz

**Affiliations:** 1 Department of Obstetric and Gynecological Nursing, Institute of Nursing and Midwifery, Medical University of Gdańsk, 80-210 Gdańsk, Poland; marzena.strahl@gumed.edu.pl; 2 Department of Allergology, Medical University of Gdańsk, 80-210 Gdańsk, Poland; ewasilewska@gumed.edu.pl; 3 Department of Preventive Medicine, Faculty of Health Sciences, Poznan University of Medical Sciences, 61-701 Poznań, Poland; 4 Department of Clinical Nutrition, Medical University of Gdańsk, 80-210 Gdańsk, Poland

**Keywords:** pregnancy, maternal nutrition, nutrition knowledge, dietary behaviours, prenatal nutrition education, maternal health, folic acid, omega-3 fatty acids, vitamin D

## Abstract

Background: Adequate nutrition during pregnancy is essential for maternal health, pregnancy outcomes, and foetal development. However, general awareness of healthy eating may not translate into detailed nutritional knowledge or appropriate dietary practices. This study assessed nutritional knowledge and selected dietary behaviours among pregnant women from urban and rural areas of the Pomeranian Voivodeship, Poland, in two periods: 2008–2010 and 2017–2019. Methods: A repeated cross-sectional questionnaire study was conducted among 938 pregnant women, including 630 respondents surveyed in 2008–2010 and 308 in 2017–2019. The original questionnaire assessed sociodemographic characteristics, nutritional knowledge, dietary behaviours, sources of information, supplementation practices, and selected health-related behaviours. Internal consistency was good (Cronbach’s alpha = 0.88). Results: Most respondents were aged 25–34 years, married, and had normal pre-pregnancy or early-pregnancy BMI. Compared with 2008–2010, women surveyed in 2017–2019 more often had higher education and more frequently reported income above the predefined questionnaire threshold. Although general awareness of healthy nutrition during pregnancy was high, detailed knowledge of key nutrients remained insufficient. Correct knowledge of the role of folic acid was reported by 58% of respondents, iron by 36%, vitamin D by 29%, iodine by 21%, and calcium by 18%. Prenatal supplement use was common, but only 39% reported supplementation based on medical recommendations. Frequently reported dietary problems included irregular meals, low fish consumption, frequent sweets intake, insufficient water intake, and low vegetable and fruit consumption. Knowledge of selected nutrient-rich foods was higher in 2017–2019, particularly for omega-3 fatty acid and vitamin D sources. Conclusions: Nutritional knowledge was heterogeneous, and practical gaps in dietary behaviours persisted, supporting the need for accessible and individualized prenatal nutrition education.

## 1. Introduction

Proper nutrition during pregnancy is one of the most important factors determining maternal health and the development of the foetus. The prenatal period is characterized by intensive metabolic, proliferative and differentiation processes that occur under conditions of particular sensitivity to environmental factors [1,2]. Nutrition is one of the key components of this environment; it may support normal foetal development, whereas deficiencies or dietary disturbances may contribute to adverse mechanisms of metabolic programming [3,4].

Insufficient intake of energy and basic macronutrients, including protein, carbohydrates and fats, as well as essential unsaturated fatty acids, may affect foetal organ development, hormonal regulation, growth and the later risk of metabolic diseases. Particular importance is attributed to DHA, which is involved in the development of the central nervous system and the retina [5,6,7].

An important element in assessing the nutritional status of pregnant women is the monitoring of gestational weight gain, because both insufficient and excessive weight gain during pregnancy are associated with an increased risk of adverse obstetric and metabolic outcomes. The recommendations of the Institute of Medicine/National Research Council and the position of the American College of Obstetricians and Gynecologists emphasize the need to individualize recommended gestational weight gain according to pre-pregnancy BMI [8,9].

The importance of prenatal nutrition is also described by concepts of developmental programming, including the thrifty phenotype hypothesis and rapid catch-up growth after a period of limited nutrient supply. These mechanisms may increase the susceptibility of offspring to obesity, insulin resistance, hypertension and lipid disorders later in life [4]. Therefore, assessing nutritional knowledge and dietary behaviours among pregnant women is important for identifying modifiable gaps that may affect both current pregnancy outcomes and long-term offspring health.

Previous studies conducted in Poland and other countries have shown that nutritional knowledge, dietary behaviours and supplementation practices among pregnant women are often insufficient and heterogeneous. Polish studies have reported inadequate dietary intake of selected nutrients, including DHA, as well as frequent use of dietary supplements, which was not always consistent with medical indications [10,11,12,13]. International studies and reviews have also indicated that pregnant women often do not fully adhere to dietary recommendations and that educational needs remain substantial [14]. Moreover, intervention studies suggest that structured nutrition education may improve dietary behaviours and adherence to recommendations during pregnancy [15]. However, most previous studies were conducted at a single time point or focused on selected aspects of maternal nutrition. Therefore, there is still a need for repeated regional studies that combine the assessment of nutritional knowledge, recognition of nutrient-rich food sources, dietary behaviours, supplementation practices, sources of information and perceived barriers to healthy eating.

Therefore, the present study aimed to assess the level of nutritional knowledge and selected dietary behaviours among pregnant women living in urban and rural areas of the Pomeranian Voivodeship in 2008–2010 and 2017–2019. In addition, selected sociodemographic characteristics of the respondents, sources of nutritional information, supplementation practices and barriers to following the principles of healthy nutrition during pregnancy were analysed.

## 2. Materials and Methods

### 2.1. Study Design and Study Population

This was a repeated cross-sectional questionnaire study was conducted in the Pomeranian Voivodeship, Poland in two independent study periods: May 2008–March 2010 and July 2017–September 2019. Pregnant women attending antenatal classes or outpatient antenatal clinics during the study periods were invited to participate.

The recruitment sites included antenatal care settings attended by women from both urban and rural areas of the Pomeranian Voivodeship. However, no formal stratified sampling by place of residence was applied.

### 2.2. Participants

Recruitment was conducted among women who presented to the participating recruitment sites and met the inclusion criteria. All eligible women who agreed to participate and provided written informed consent completed the questionnaire. Thus, recruitment was based on consecutive enrolment of consenting eligible women attending the participating antenatal clinics or antenatal classes during the study periods.

For the purposes of this study, “eligible women” were defined as pregnant women attending the participating recruitment sites who received information about the study, met the inclusion criteria, and were able to complete the questionnaire independently. “Participated” refers to women who agreed to take part, provided written informed consent, and completed the questionnaire. “Included in the analysis” refers to participants whose questionnaires were sufficiently complete for statistical analysis.

The inclusion criteria were: being pregnant at the time of the survey, attending an antenatal clinic or antenatal class at one of the participating recruitment sites, receiving information about the study, providing written informed consent, and agreeing to complete an anonymous questionnaire. The exclusion criteria were: lack of consent, inability or unwillingness to complete the questionnaire, and incomplete questionnaires that did not allow inclusion in the final analysis.

Recruitment was conducted by nurses or midwives involved in antenatal care. Eligible women were informed about the aim of the study, its voluntary and anonymous nature, and the use of the collected data exclusively for scientific purposes. Each participant received written information about the study and an informed consent form. After signing the consent form, participants completed the anonymous questionnaire independently and returned it to the nurse or midwife.

### 2.3. Data Collection and Research Tool

The research tool was an original questionnaire prepared at the Department of Obstetrics and Gynaecological Nursing, Chair of Nursing, Medical University of Gdansk. The questionnaire was developed on the basis of a literature review concerning nutritional knowledge and behaviours during pregnancy, educational materials from the programme “I can already take care of the health of my future child”, and the 5 A’s protocol [10].

Before the main study, the questionnaire was pilot-tested in a group of pregnant women to assess the clarity, comprehensibility, and practical feasibility of the questions. After the pilot phase, the questionnaire was used in the main survey.

The questionnaire contained 55 questions, including 2 open-ended questions, 13 semi-open questions, 28 closed-ended questions, and a demographic section consisting of 12 questions. The questions concerned knowledge of the role of vitamins and other dietary components, health-promoting behaviours during the reproductive period and pregnancy, dietary habits, alcohol consumption, and tobacco smoking.

The reliability of the questionnaire was assessed using Cronbach’s alpha coefficient, which was 0.88, indicating good internal consistency of the tool.

### 2.4. Variables and Measurement

Dietary behaviour indicators were derived from self-reported questionnaire items. Meal regularity was assessed using questions on the number of meals consumed per day and the reported regularity of individual meals. Responses indicating irregular meal consumption were classified as an irregular meal pattern.

The frequency of consumption of selected food groups was assessed using predefined questionnaire response categories, including “daily”, “several times per week”, “several times per month”, “never”, and “depending on financial possibilities”. Low consumption of vegetables and fruit, as well as fish, was defined as reporting consumption “several times per month” or “never”. Frequent sweets intake was defined as reporting sweets consumption “daily” or “several times per week”.

### 2.5. Study Size and Participant Flow

**Figure 1:**
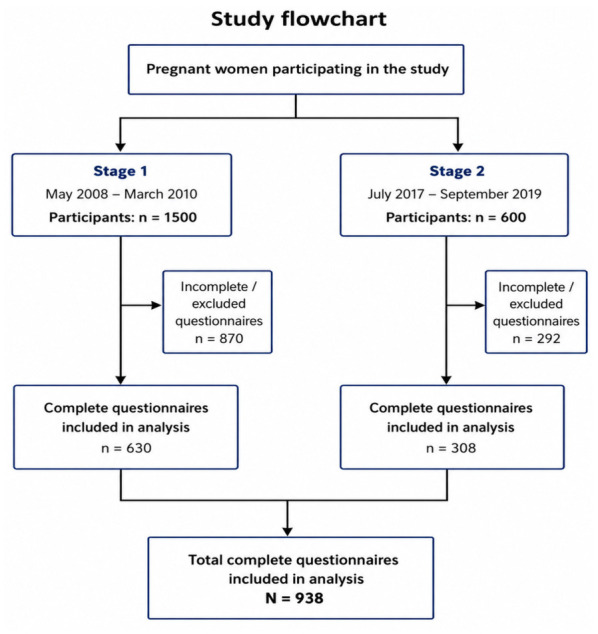
Flowchart of participant recruitment and inclusion in the final analysis across the two study periods.

A total of 1500 women completed the questionnaire in 2008–2010, and 600 women completed the questionnaire in 2017–2019. After exclusion of incomplete questionnaires, 938 questionnaires were included in the final statistical analysis, 630 from 2008–2010 and 308 from 2017–2019. The proportions of questionnaires included in the analysis were therefore 42.0% and 51.3%, respectively. The participant flow is presented in Figure 1.

### 2.6. Bias

The proportion of questionnaires not included in the final analysis was high in both study periods. In 2008–2010, 870 of 1500 returned questionnaires were incomplete or excluded, whereas in 2017–2019, 292 of 600 returned questionnaires were incomplete or excluded. The reasons for incompleteness and the missingness pattern for all key variables could not be fully reconstructed from the archived dataset. Therefore, the possibility of selection bias related to questionnaire completeness should be considered when interpreting the findings.

In addition, because recruitment was conducted among women attending participating antenatal clinics or antenatal classes and no formal stratified sampling by place of residence was applied, the study sample may not fully represent all pregnant women in the Pomeranian Voivodeship.

### 2.7. Ethical Considerations

Participants were informed about the voluntary and anonymous nature of the study, and the data obtained were used only for scientific purposes. The study was approved by the Bioethical Committee of medical University of Gdansk (approval no. NKEBN/160/2007).

### 2.8. Statistical Analysis

The analyses were conducted using Microsoft Excel, Stat Soft STATISTICA 14.1.0, and the R environment. The basic analyses were performed in R version 4.1.0, whereas supplementary and verification analyses were carried out in R version 4.4.1.

Quantitative variables were described using the arithmetic mean, median, standard deviation, and minimum and maximum values. Qualitative variables were presented as absolute numbers (*n*) and percentages (%).

The selection of statistical tests depended on the type of variables, the number of groups compared, and whether the assumptions for individual tests were met. Associations between qualitative variables were assessed using Pearson’s chi-square test, while Fisher’s exact test was applied in cases of small expected cell counts. Comparisons involving more than two independent groups for ordinal variables or quantitative variables with non-normal distributions were performed using the Kruskal–Wallis test. Associations between quantitative variables were assessed using Pearson’s correlation coefficient; in analyses that did not meet parametric assumptions, the appropriate rank correlation coefficient was interpreted.

For dichotomous comparisons, the effect size measure phi (φ) was also calculated, and its values were interpreted as the strength of association independently of the statistical significance level. A *p*-value of <0.05 was considered statistically significant. The results are presented in tables and figures.

## 3. Results

### 3.1. Characteristics of the Study Group

The largest age groups were women aged 25–29 years (34.01%) and 30–34 years (30.81%). Most respondents were in the second or third trimester of pregnancy. More than half of the women had no children (53.84%), and 29.10% had one child. Normal BMI before pregnancy or at the beginning of pregnancy was reported by 70.68% of women. Most respondents were married (82.62%).

In the whole group, the largest proportion of women had secondary education (41.90%) or higher education (30.60%). Compared with the first study period, the proportion of women with higher education increased markedly from 17.62% in 2008–2010 to 57.14% in 2017–2019. At the same time, the proportions of women with vocational and primary education decreased. It should be noted that the number of respondents differed between the two study periods. The final analytical sample included 630 women surveyed in 2008–2010 and 308 women surveyed in 2017–2019. Therefore, comparisons between the two periods should be interpreted with caution, particularly because the unequal group sizes may influence the precision of estimates and the stability of between-period comparisons. The results are presented in Table 1.

### 3.2. Sociodemographic Changes Between the Study Periods

The sociodemographic profile of the respondents differed between the two study periods. The proportion of women with higher education increased, and a higher proportion of respondents in 2017–2019 reported income above the predefined threshold of 1000 PLN per family member compared with women surveyed in 2008–2010. This threshold was a self-reported income category used in the questionnaire; therefore, because the same nominal cut-off was applied in both study periods, it should be interpreted descriptively and not as a directly comparable measure of purchasing power or socioeconomic status over time.

The age structure shifted towards the 25–29-year age group, which accounted for 40.91% of respondents in 2017–2019. A slight increase in the proportion of women aged 35–39 years was also observed, along with a decrease in the proportions of women in the extreme age groups: 16–18 years and 40–43 years. In 2017–2019, women in the third trimester of pregnancy clearly predominated across all places of residence.

### 3.3. Level of Nutritional Knowledge

General awareness of the importance of proper nutrition during pregnancy was relatively high; most women declared that diet during pregnancy has an important impact on the health of the mother and child. At the same time, detailed knowledge of the components of a balanced diet was incomplete. Overall, 84% of respondents recognized the general importance of healthy eating, whereas only 41% were able to correctly identify specific components of a balanced prenatal diet.

**Figure 2:**
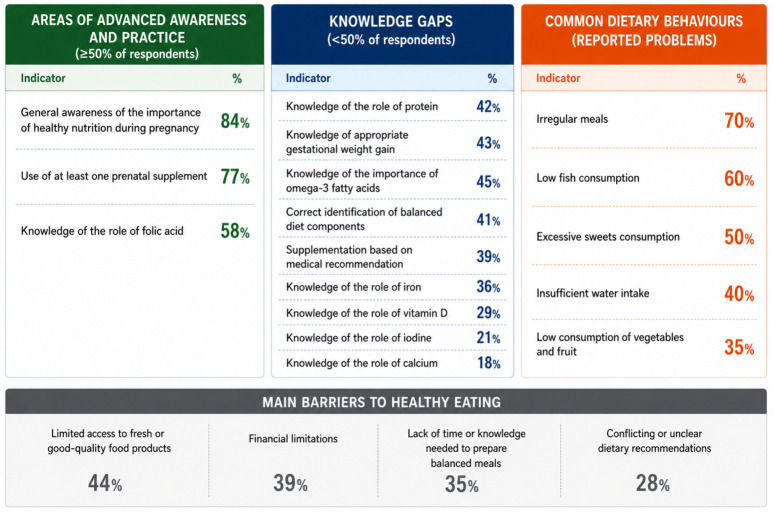
Self-reported nutritional knowledge, dietary behaviour indicators, and perceived barriers to healthy eating among pregnant women in the study population. Values represent the percentage of respondents reporting each characteristic. Dietary behaviour indicators were derived from questionnaire response categories and should not be interpreted as quantitative measures of food, nutrient, or fluid intake.

As summarized in Figure 2, although general awareness of healthy nutrition during pregnancy was high, and prenatal supplementation was common, important gaps remained in knowledge of specific nutrients, and several unhealthy dietary behaviours were frequently reported.

The greatest knowledge gaps concerned the role of protein, appropriate gestational weight gain, the importance of omega-3 fatty acids and principles of meal planning, such as regularity and variety. Knowledge of selected vitamins and minerals varied; the role of folic acid was correctly recognized by 58% of respondents, iron by 36%, vitamin D by 29%, iodine by 21% and calcium by 18%.

### 3.4. Dietary Behaviours and Sources of Information

Some women reported improving their diet after pregnancy was confirmed, including limiting fast food, increasing vegetable consumption and eating meals more regularly. Nevertheless, dietary irregularities were still observed. The most frequently reported problems were irregular meals, low fish consumption, excessive intake of sweets, insufficient water intake and low consumption of vegetables and fruit (Figure 2).

The main sources of nutritional information were midwives and gynaecologists, the Internet, social media and family. Although more than half of the women indicated medical personnel as a source of knowledge, only some respondents received structured nutritional counselling. A considerable proportion used non-professional sources of information, which may contribute to misinterpretation of recommendations or self-selection of supplements.

The most frequently reported barriers to healthy eating were limited access to fresh or good-quality food products (44%), financial limitations (39%), lack of time or knowledge required to prepare balanced meals (35%), and conflicting or unclear dietary recommendations (28%).

### 3.5. Use of Stimulants During the Preconception Period and Pregnancy

Risk behaviours persisted in some women. Approximately 25% of respondents smoked tobacco in the preconception period, and about 12% reported alcohol consumption during the first weeks of pregnancy. These findings suggest that health education should cover not only pregnancy itself, but also the period of pregnancy planning.

### 3.6. Nutritional Knowledge Regarding Selected Nutrient Sources

**Figure 3:**
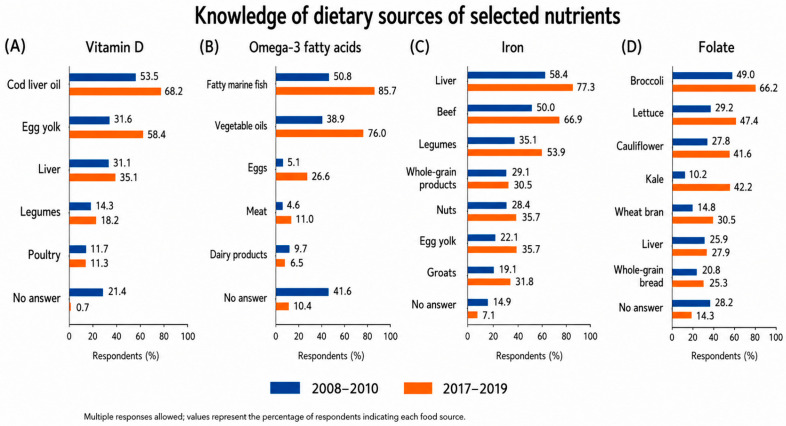
Knowledge of dietary sources of vitamin D (**A**), omega-3 fatty acids (**B**), iron (**C**), and folate (**D**) among pregnant women in 2008–2010 and 2017–2019. Values represent the percentage of respondents indicating each food source.

The respondents’ knowledge of selected nutrient-rich food products varied across nutrients and changed between the two study periods. Overall, the 2017–2019 group more frequently identified key dietary sources of folate, iron, omega-3 fatty acids and vitamin D than the 2008–2010 group. The largest between-period difference was observed for the identification of omega-3 fatty acid sources, while women surveyed in 2017–2019 also more frequently identified several folate- and vitamin D-rich food items than women surveyed in 2008–2010 (Figure 3).

As shown in Figure 3, the proportion of women who correctly identified selected folate-rich foods increased in the later study period. Broccoli was the most frequently indicated source of folate in both periods, with an increase from 49.05% in 2008–2010 to 66.23% in 2017–2019. The percentage of respondents indicating lettuce increased from 29.21% to 47.40%, cauliflower from 27.78% to 41.56%, and kale from 10.16% to 42.21%. At the same time, the proportion of women who did not provide an answer decreased from 28.25% to 14.29%.

Knowledge of dietary iron sources also changed in the later study period (Figure 3C). Liver was the most frequently indicated iron-rich product, with an increase from 58.41% to 77.27%. The proportion of respondents indicating beef increased from 50.00% to 66.88%, while indications of legumes increased from 35.08% to 53.90%. A decrease in the “no answer” category was also observed, from 14.90% to 7.14%, suggesting better recognition of iron-containing foods among women surveyed in 2017–2019.

The largest between-period difference was observed in the identification of omega-3 fatty acid sources (Figure 3B). The proportion of women indicating fatty marine fish increased markedly from 50.79% in 2008–2010 to 85.71% in 2017–2019. Similarly, indications of selected vegetable oils increased from 38.89% to 75.97%. In parallel, the percentage of respondents who did not answer this question decreased substantially, from 41.59% to 10.39%.

Knowledge of dietary vitamin D sources was also higher in the 2017–2019 group (Figure 3A). Cod liver oil was the most frequently indicated source in both study periods, increasing from 53.49% to 68.18%. A particularly large increase was observed for egg yolk; the proportion of responses indicating this product rose from 31.59% in 2008–2010 to 58.44% in 2017–2019. The proportion of non-responses decreased from 21.43% to 0.65%, indicating a substantial reduction in non-response for this item.

Taken together, these findings indicate higher reported identification of selected nutrient-rich products among women surveyed in 2017–2019 compared with those surveyed in 2008–2010. However, despite this positive trend, knowledge remained incomplete for several food groups, particularly in relation to less commonly recognized sources of key nutrients. This suggests that nutrition education in pregnancy should not only emphasize the importance of supplementation but also provide practical guidance on dietary sources of essential nutrients.

In addition to improved recognition of individual food sources, the ability to correctly identify selected nutrient-food source pairs also increased between the two study periods. The largest changes were observed for omega-3 fatty acid and vitamin D source identification, with smaller changes noted for selected folate- and iron-related food pairs. Between-period differences in the identification of selected nutrient-rich products are presented in Figure 3 and Table 2.

Statistically significant differences according to place of residence were found for folate-rich products, iron-rich products, and vitamin D sources: the percentage of indications was highest among respondents from rural areas for folate-rich products and vitamin D sources, while for iron-rich products it was slightly highest among women from cities with fewer than 50,000 inhabitants; no significant differences were found for omega-3 fatty acid sources (Table 3).

### 3.7. Prenatal Supplementation

Prenatal use of vitamin and mineral preparations was reported more frequently in 2017–2019 than in 2008–2010, 89.6% vs. 72.7%, respectively. This between-period difference was statistically significant: χ^2^ (2) = 36.84, *p* < 0.001, Cramér’s V = 0.198. Overall, 39% of respondents reported that supplementation had been recommended by a physician, midwife, or other healthcare professional. However, stratified data on medically recommended supplementation by study period were not available in the archived dataset. The available data also did not allow a detailed assessment of supplement type, dose, timing of initiation, duration of use, or adherence to nutrient-specific recommendations.

## 4. Discussion

The present study showed that general awareness of the importance of proper nutrition during pregnancy does not necessarily translate into detailed nutritional knowledge or appropriate dietary behaviours. Although most respondents recognized the relevance of diet for maternal and foetal health, their understanding of the components of a balanced prenatal diet and the role of key nutrients remained limited, particularly with regard to protein, omega-3 fatty acids, iron, vitamin D, iodine, and calcium. This limited awareness is clinically relevant, as these nutrients play essential roles in foetal growth, nervous system development, haematopoiesis, bone mineralization, thyroid function, and immunity. Insufficient knowledge of their importance may contribute to inadequate dietary intake, inappropriate supplementation, or difficulties in implementing dietary recommendations in everyday practice. Importantly, these shortcomings persisted despite the more favourable sociodemographic profile of women surveyed in 2017–2019, including higher educational attainment and better self-reported material status compared with those surveyed in 2008–2010.

Our findings suggest that differences in sociodemographic characteristics and access to health-related information may not fully explain the observed gaps in nutrition literacy and dietary behaviours. The discrepancy between high general awareness and persistent dietary irregularities suggests that nutritional knowledge may remain largely declarative and may not be sufficient to guide everyday food choices. Pregnant women may recognize that healthy eating is important, but still lack the practical nutritional literacy needed to identify nutrient-rich foods, plan regular meals, and apply dietary recommendations in real-life conditions. This gap may be reinforced by reliance on non-professional information sources, conflicting dietary messages, financial limitations, limited access to fresh or good-quality food products, and insufficient individualized counselling by healthcare professionals. Therefore, the observed dietary behaviours should not be interpreted only as a consequence of inadequate knowledge but rather as the result of interacting educational, informational, socioeconomic, and practical barriers. These findings support the need for structured prenatal nutrition education focused not only on general awareness but also on practical implementation of recommendations in daily life. Given the key role of nurses and midwives in routine prenatal care, structured nutrition education delivered by these professionals may be particularly important for translating general awareness into practical dietary behaviours [11,12].

The persistence of inadequate dietary behaviours may also be interpreted in the context of health and nutritional literacy. Access to information alone does not ensure that pregnant women are able to understand, critically evaluate, and apply dietary recommendations in everyday life. Recent studies have emphasized that digital and e-health literacy may influence sustainable nutrition behaviours in pregnant women, while discrepancies in micronutrient knowledge and supplementation practices between pregnant women and healthcare providers may further complicate the implementation of nutritional recommendations [11,12]. This is particularly important in the current information environment, where social media, websites, online forums, and informal advice increasingly shape perceptions of healthy eating during pregnancy. Although these sources may improve access to information, they may also disseminate simplified, inconsistent, or non-evidence-based messages, including misinformation and fake news, which may lead to confusion, unnecessary dietary restrictions, inappropriate supplementation, or reduced trust in professional recommendations.

At the same time, dietary behaviours are influenced not only by individual knowledge, but also by structural barriers such as financial limitations, limited access to fresh and good-quality food products, transportation difficulties, time constraints, and unequal access to professional nutritional counselling. Although the data analysed in the present study were collected before the COVID-19 pandemic and therefore cannot directly assess its impact, future studies should consider whether pandemic-related restrictions, economic insecurity, reduced access to healthcare, and increased reliance on online information further modified eating behaviours and sources of nutritional advice during pregnancy.

The value of the present study lies in its repeated cross-sectional design, which enabled the comparison of nutritional knowledge and selected dietary behaviours among pregnant women across two distinct time periods characterized by changes in educational attainment, socioeconomic conditions, and access to health information.

The observed sociodemographic differences may partly explain variation in access to reliable nutrition information and in the practical ability to follow dietary recommendations, particularly because education, place of residence, and self-reported material status can influence both health literacy and food choices. The higher proportion of women with university education in the 2017–2019 sample should not be interpreted as direct evidence of improved education in the underlying population, because the two study periods included independent samples with different structures. Nevertheless, this finding suggests that formal education alone may not ensure pregnancy-specific nutritional literacy or the practical ability to translate dietary recommendations into everyday behaviours. Women with higher education and those living in larger cities may have greater access to professional counselling and healthcare services, whereas women living in rural areas and small towns may face barriers related to counselling availability, transport, food costs, and established dietary patterns [10,13,14,15]. Therefore, educational and socioeconomic progress should be accompanied by targeted, practical, and professionally guided nutrition education aimed at translating knowledge into everyday dietary behaviours.

The 2008–2010 and 2017–2019 samples differed in educational level and in the proportion of respondents reporting income above the predefined questionnaire threshold. However, because this was a fixed nominal income category, it should not be interpreted as a directly comparable measure of material status across the two periods. At the same time, the persistence of dietary irregularities, such as irregular meals, low fish intake and excessive consumption of sweets suggest that access to information alone is not sufficient. Practical, consistent and repeated educational activities delivered by medical personnel should be considered [16,17].

Our results are consistent with the broader picture of nutritional problems observed among pregnant women in Poland. The importance of health education was also confirmed by Demuth et al., who showed that previous health education was significantly associated with better diet quality among pregnant women in Poland. In that study, women with a history of health education had more than a threefold higher chance of better diet quality, although even in this group the health-promoting properties of the diet remained unsatisfactory [18]. These findings suggest that nutritional awareness is important but insufficient without practical, repeated support in implementing recommendations.

Similar conclusions can be drawn from the comparative analysis of nutrient intake among pregnant women in Spain and Poland conducted by Iglesias-Vázquez et al. The authors showed that, despite cultural and dietary differences between countries, inadequate intakes of iron, vitamin D and folates/vitamin B9 were observed in both populations [19]. In the Polish population, differences in the intake of macronutrients and selected vitamins were also observed, which indicates the need for tailored nutritional counselling rather than only general prenatal recommendations [20].

Insufficient implementation of dietary recommendations was also confirmed in a large cohort study by Jankowska et al. involving women from the Polish Mother and Child Cohort. The authors assessed the intake of selected minerals and vitamins and their blood concentrations. Most women did not meet the requirements for the analysed nutrients from diet alone, despite the fact that approximately 94% reported using supplements [20]. This study shows that supplementation is common but does not always indicate actual coverage of nutritional needs, and that the risk of deficiency depends, among other factors, on previous diet, physical activity, BMI, stress level and the timing of the initiation of medical care.

DHA is a particularly important example of insufficient intake of a nutrient that is essential for foetal development. Wierzejska et al. demonstrated very low dietary DHA intake among pregnant women; the median intake was 60 mg/day, and after including supplements it was 90 mg/day, with only 28% of women using DHA preparations [21]. The authors indicated that the diet of pregnant women was largely deficient in DHA and that achieving current recommendations without supplementation is unlikely.

At the same time, the most recent data from Brodziak-Dopierała et al. show that the use of dietary supplements by pregnant women in Poland is very common. More than 90% of respondents reported supplementation during pregnancy, most often with multicomponent preparations, and the most frequently used ingredients were folic acid and DHA, followed by iron, iodine, magnesium and calcium [22]. In only about half of the women was the decision to use supplementation consistent with medical indications, which indicates the need to individualize recommendations and monitor the quality of counselling.

Taken together, the cited studies indicate that the problem of nutrition among pregnant women in Poland is not limited to a single deficiency but involves a broader set of relationships: level of knowledge, diet quality, adequacy of micronutrient intake, supplement use and access to professional counselling [20,21,22,23]. The findings of the present study should therefore be interpreted in the context of the need for more systematic, practical and personalized nutrition education during pregnancy, covering both diet and rational supplementation.

The importance of nutrition education during pregnancy is also supported by recent international data. Olloqui-Mundet et al., in a review of dietary habits, nutritional knowledge and perceptions of nutrition education among pregnant women, indicated that an appropriate diet during pregnancy is important for maternal and foetal health, but many studies still report irregularities, including excessive gestational weight gain and insufficient adherence to dietary recommendations [24]. The authors emphasized the need to identify educational gaps and adapt interventions to the actual needs of pregnant women, which is consistent with the Polish studies discussed earlier.

The practical importance of nutrition education is also demonstrated by the randomized study by Goodarzi-Khoigani et al., in which an educational intervention based on Pender’s Health Promotion Model improved the diet of pregnant women and their adherence to dietary recommendations [25]. These results indicate that education should not be merely informational but should be a planned behavioural intervention that takes into account beliefs, motivation and practical possibilities for behaviour change.

The findings of the present study may also be interpreted in the broader context of health-related behaviours among pregnant women. Boguszewski et al., in a study conducted in Warsaw, showed that pregnant women declared greater attention to a healthy lifestyle than non-pregnant women, and that a higher level of health-promoting behaviours was associated, among other factors, with physical activity [26]. The authors suggest that pregnancy may be a period of increased readiness for change and greater interest in one’s own health, representing an important “window of opportunity” for educational and preventive activities.

Although tobacco smoking and alcohol consumption were not the primary focus of the present analysis, they provide additional context for interpreting nutrition-related behaviours as part of a broader pattern of health behaviours during pregnancy. At the same time, pregnancy itself does not eliminate risk behaviours. Balwicki et al. showed that among pregnant women from small towns in Poland, 34.6% reported smoking at the beginning of pregnancy; 14.7% quit smoking during pregnancy, whereas 19.9% continued to smoke [27]. These data show that even during a period of high health motivation, some women require more intensive and targeted support in changing behaviours, not only information about risk.

Another analysis by Balwicki et al. concerning smoking women from small towns and rural areas in Poland indicated that smoking cessation during pregnancy depends on many social and behavioural factors [28]. In the context of the present study, this strengthens the conclusion that effective prevention during pregnancy should include not only nutrition education but also an assessment of the broader profile of health behaviours, environmental barriers and the patient’s readiness to change.

The findings concerning tobacco smoking and alcohol consumption confirm the need to start education before pregnancy. The early period of pregnancy, which often precedes its recognition, is particularly sensitive to risk factors. Therefore, counselling should include women of reproductive age, not only patients already receiving prenatal care [27,28].

## 5. Limitations of the Study

Several limitations should be considered when interpreting the findings of this study. First, the study was conducted in a single region of Poland, the Pomeranian Voivodeship, which may limit the generalizability of the results to pregnant women from other regions or countries.

Second, although the study included a relatively large sample, the participants were recruited from antenatal classes and outpatient antenatal clinics, which may have introduced selection bias. Women attending these settings may be more health-conscious and more interested in pregnancy-related education than the general population of pregnant women.

Third, the data were collected using a self-administered questionnaire; therefore, the results may be affected by recall bias and social desirability bias. Respondents may have overreported healthy behaviours or underestimated behaviours perceived as unfavourable, such as irregular meals, low intake of recommended products, alcohol consumption, or smoking. In addition, the assessment focused on declared nutritional knowledge and self-reported dietary behaviours and did not include objective measures of dietary intake, such as dietary recalls, food records, validated food frequency questionnaires, or nutritional biomarkers. Therefore, the reported dietary behaviours should not be interpreted as precise quantitative measures of food, nutrient, or fluid intake.

Fourth, the questionnaire used in the study was an original research tool. Although it was pilot-tested before the main study and demonstrated good internal consistency, it was not a widely standardized instrument. This should be taken into account when comparing the results with other studies.

Fifth, the two study periods differed in several sociodemographic and pregnancy-related characteristics, including educational level, self-reported material status, and trimester of pregnancy. In addition, the number of respondents differed between the two study periods, with 630 women included in 2008–2010 and 308 in 2017–2019. These differences may have influenced the precision of estimates and the observed changes between the two periods, and they should therefore be considered when interpreting time-related comparisons. These differences may have influenced the precision of estimates and the observed changes between two periods and they should be therefore considered when interpreting time-related comparisons. Moreover, changes in access to health information, including the increasing role of the Internet and social media during the study period, may have affected women’s sources of nutritional knowledge.

Finally, due to the repeated cross-sectional design, causal relationships between sociodemographic factors, nutritional knowledge, and dietary behaviours cannot be established. The study allows for the identification of associations and differences between the two periods, but it does not permit conclusions regarding individual changes over time.

## 6. Conclusions

1. Among pregnant women surveyed in the Pomeranian Voivodeship in 2008–2010 and 2017–2019, nutritional knowledge was heterogeneous and appeared to vary according to selected sociodemographic characteristics, including education, place of residence and material status.
2. Despite high general awareness of the importance of healthy nutrition during pregnancy, many women show gaps in detailed knowledge concerning the components of a balanced diet and the role of protein, omega-3 fatty acids, vitamins and minerals.
3. Self-reported dietary behaviours remained suboptimal in some respondents, particularly with regard to meal regularity, fish consumption, intake of vegetables and fruit, and limiting sweets.
4. The observed gaps in practical nutritional knowledge and dietary behaviours support the need for systematic, accessible and individualized prenatal nutrition education integrated into routine prenatal care, including evidence-based digital strategies and targeted support for women with limited access to reliable nutritional information.
5. Due to the repeated cross-sectional design, unequal sample sizes, differences in sample composition and reliance on self-reported data, the findings should be interpreted cautiously and should not be considered evidence of causal relationships.

## 7. Future Perspectives

Future studies should use validated nutritional literacy tools and objective methods of dietary assessment, such as food frequency questionnaires, dietary recalls, food records and biochemical markers. Longitudinal and multicentre studies are needed to assess changes in nutritional knowledge during pregnancy and to improve the generalizability of findings. Further research should also evaluate personalized and digital prenatal nutrition education interventions integrated into routine prenatal care.

## Figures and Tables

**Table 1:** Selected sociodemographic and pregnancy-related characteristics of the study population. Data are presented as *n* (%).

Age 25–29 years	193 (30.63%)	126 (40.91%)	319 (34.01%)
Age 30–34 years	203 (32.22%)	86 (27.92%)	289 (30.81%)
Second trimester of pregnancy	337 (53.49%)	58 (18.83%)	395 (42.11%)
Third trimester of pregnancy	127 (20.16%)	230 (74.68%)	357 (38.06%)
No children	333 (52.86%)	172 (55.84%)	505 (53.84%)
BMI (body mass index) 18.5–24.9	445 (72.59%)	218 (70.78%)	663 (70.68%)
Income > 1000 PLN per person *	181 (28.73%)	204 (66.23%)	385 (41.04%)
Married	503 (79.84%)	272 (88.31%)	775 (82.62%)

* Income > 1000 PLN per person was a predefined self-reported income category used in the questionnaire. Because the same absolute threshold was applied in both study periods, it should be interpreted only descriptively and not as a directly comparable measure of purchasing power or socioeconomic status over time.

**Table 2:** Statistical significance of differences between the study periods for selected indications of nutrient-rich products.

Nutrient/ Item	Product/ Response	2008–2010 (%)	2017–2019 (%)	*p*-Value	Phi (φ)
Folate	Broccoli	49.05	66.23	<0.001	0.162
	Lettuce	29.21	47.40	<0.001	0.179
	Cauliflower	27.78	41.56	<0.001	0.138
	Kale	10.16	42.21	<0.001	0.372
	No answer	28.25	14.29	<0.001	0.154
Iron	Liver	58.41	77.27	<0.001	0.185
	Beef	50.00	66.88	<0.001	0.160
	Legumes	35.08	53.90	<0.001	0.179
	No answer	14.90	7.14	0.001	0.111
Omega-3 fatty acids	Fatty marine fish	50.79	85.71	<0.001	0.338
	Vegetable oils	38.89	75.97	<0.001	0.348
	No answer	41.59	10.39	<0.001	0.316
Vitamin D	Cod liver oil	53.49	68.18	<0.001	0.140
	Egg yolk	31.59	58.44	<0.001	0.257
	No answer	21.43	0.65	<0.001	0.276

*p*-values were calculated for comparisons between 2008–2010 (*n* = 630) and 2017–2019 (*n* = 308). Phi (φ) was used as the effect-size measure for 2 × 2 comparisons. Because participants could indicate more than one product, percentages within each nutrient category do not sum to 100%.

**Table 3:** Association between place of residence and knowledge of selected nutrient-rich food products in the total sample.

Nutrient-Source Category	Cities > 50,000 *n* (%)	Cities < 50,000 *n* (%)	Rural Areas *n* (%)	Total N	*p*-Value	χ^2^ (df)	Cramér’s V
**Folate-rich products**	958 (31.5)	1033 (33.9)	1052 (34.6)	3043	0.032	45.82 (30)	0.087
**Iron-rich products**	1148 (32.9)	1183 (33.9)	1163 (33.3)	3494	<0.001	70.04 (32)	0.100
**Omega-3 fatty acid sources**	569 (32.6)	587 (33.7)	587 (33.7)	1743	0.093	31.17 (22)	0.095
**Vitamin D sources**	596 (31.1)	638 (33.3)	680 (35.5)	1914	0.004	40.69 (20)	0.103

Data are presented as *n* (%) of responses by place of residence. Percentages are row percentages and may not sum to exactly 100% due to rounding. Total N refers to the total number of responses, not the number of individual respondents, because participants could indicate more than one product. Associations were assessed using the χ^2^ test for contingency tables, and effect size was reported as Cramér’s V. Statistical significance was set at *p* < 0.05.

## Data Availability

The dataset is available from the corresponding author upon reasonable request.

## Abbreviations

The following abbreviations are used in this manuscript:

ACOG	American College of Obstetricians and Gynecologists
BMI	Body Mass Index
DHA	Docosahexaenoic Acid
IOM	Institute of Medicine
NRC	National Research Council
PLN	Polish Złoty
WHO	World Health Organization
